# 2-Hydroxypropyl-beta-cyclodextrin (HPβCD) reduces age-related lipofuscin accumulation through a cholesterol-associated pathway

**DOI:** 10.1038/s41598-017-02387-8

**Published:** 2017-05-19

**Authors:** Jason Gaspar, Jacques Mathieu, Pedro Alvarez

**Affiliations:** Rice University, Dept of Civil and Environmental Engineering, MS-6398, 6100 Main Street, Houston, TX 77005 USA

## Abstract

Oxidative stress causes significant increases in both cholesterol uptake and intracellular accumulation of the aging biomarker lipofuscin. Here we show that HPβCD addition mitigates these adverse effects in human fibroblasts by significantly reducing *LDLr* and *SREBP1* gene expression. In the absence of oxidative stress, HPβCD addition induces a paradoxical response, increasing cholesterol accumulation (but not lipofuscin) via upregulation of cholesterol biosynthesis. These two distinct, but opposite effects highlight a previously overlooked therapeutic consideration: the cholesterol content of the treated cell determines which cholesterol pathways, either beneficial or harmful, are responsive to HPβCD.

## Introduction

The sterol binding compound, 2-Hydroxypropyl-beta-cyclodextrin (HPβCD), has shown promise in animal studies for treating Alzheimer’s disease, Niemann-Pick disease (NPC), age-related macular degeneration, and atherosclerosis^1–5^. However, HPβCD’s mode of action remains elusive, and the scientific community is divided into two main camps: (1) those who hypothesize that HPβCD nonspecifically binds and liberates cholesterol and oxysterols, thus shifting overall cellular cholesterol equilibrium away from the cell, and (2) those who postulate that HPβCD transports sequestered cholesterol from the lysosome to the cytosol like the NPC1 and NPC2 proteins^6^.

Recognizing that the above age-related disorders have well established links to cholesterol processing and membrane biology^7^, here we investigate whether HPβCD could also be an effective therapeutic to remove the aging biomarker lipofuscin (LF). LF is an autofluorescent polymeric amalgam with significant lipid content, which accumulates within postmitotic cells during aging^8^. LF accumulation has an inverse relationship with lifespan, impairing proteosome and lysosome functions critical to cell health and homeostasis^9^. Furthermore, LF is observed in each of the above diseases^10–14^, often in cells with perturbed cholesterol homeostasis^15^. LF clearance by HPβCD would further validate HPβCD’s therapeutic potential as well as provide insights into HPβCD’s therapeutic mechanisms.

Here, we resolve the long-standing debate about HPβCD’s mode of action and report a previously overlooked potential adverse effect of HPβCD-based treatment. Specifically, we show that the cholesterol content of treated cells determines HPβCD’s therapeutic efficacy versus possible unintended harm.

## Results

**Figure 1:**
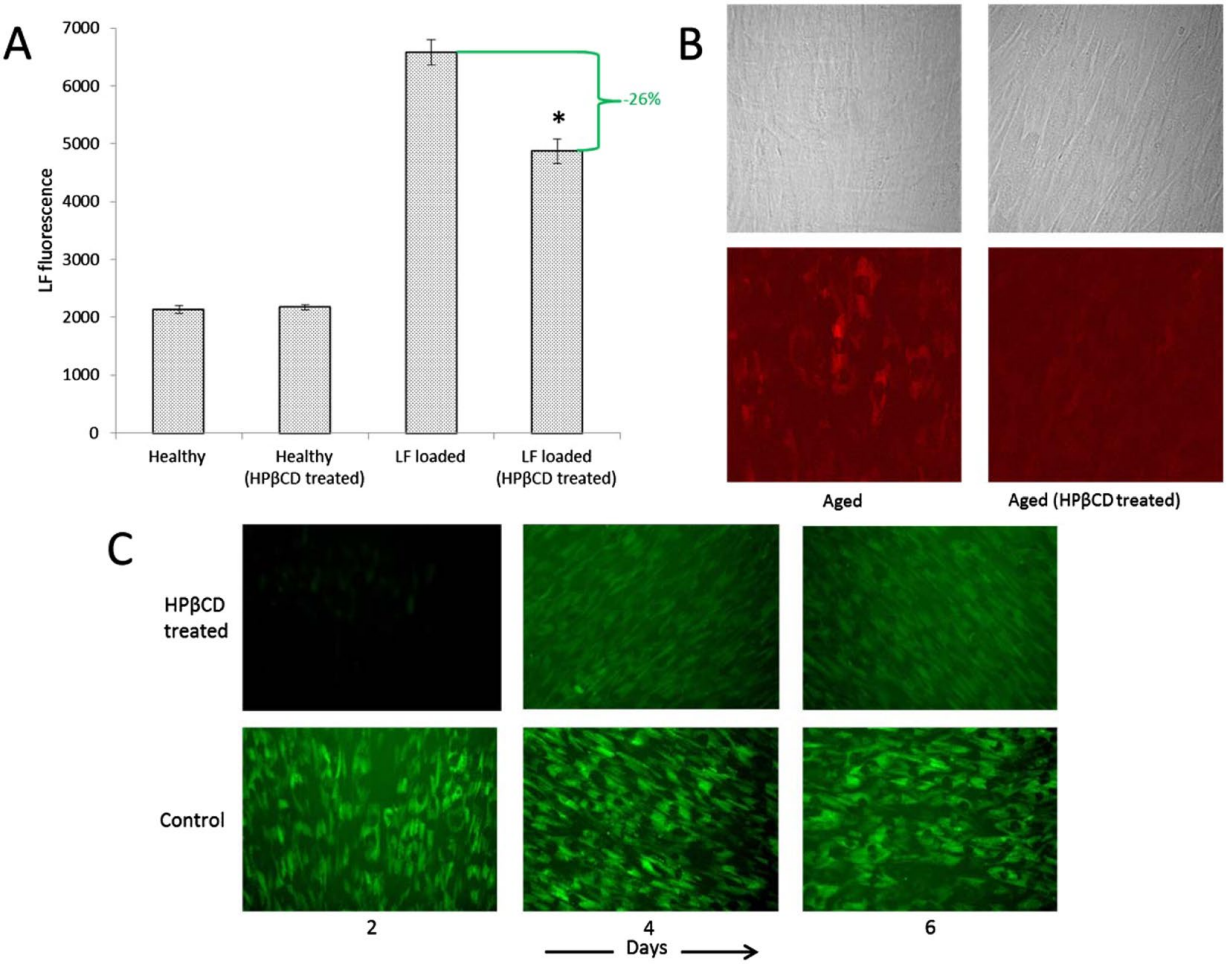
HPβCD reduces LF and lipopigment. (**A**) Exposure of skin fibroblasts to oxidizing conditions for 10 days loads cells with LF. HPβCD addition on days 5–10 reduces LF totals by 26%. Asterisk (*) indicates statistically significant reduction (*p* < 0.001, N = 4). (**B**) Serial passaged fibroblasts accumulate LF (left column). HPβCD treatment significantly reduces LF accumulation (right column). Light microscopy (top row) and LF imaging (bottom row) (**C**) HPβCD slows lipopigment accumulation. Healthy human skin fibroblasts were exposed to oxidizing conditions for 8 continuous days in the presence and absence of HPβCD.

HPβCD addition to aged LF-loaded, but otherwise healthy, human skin fibroblasts significantly reduced lipofuscin levels (−26%, p < 0.001, Fig. 1A). HPβCD was also added to fibroblasts that had been serially passaged to induce an aging phenotype with visual LF present before HPβCD treatment. Figure 1B demonstrates the ability of HPβCD to remove preexisting LF. We also exposed healthy unaged fibroblasts to oxidizing conditions that induce LF and lipopigment accumulation. In the presence of HPβCD, lipopigment accumulation was visually slowed and total accumulation decreased (Fig. 1C).

To determine HPβCD’s mode of action, we first considered the possibility that HPβCD upregulates lysosome biogenesis which subsequently leads to LF exocytosis. The lysosomal biogenesis regulatory gene, *TFEB*, modulates intracellular Ca^2+^ levels, and has been observed to be overexpressed upon HPβCD addition^16, 17^. Also, LF has been reported to accumulate within cell lysosomes^8^. For our system, however, *TFEB* upregulation was not observed at multiple time points (1 hour or 4 and 10 days) after HPβCD treatment (Fig. S1). The absence of TFEB upregulation indicates that TFEB activation did not confound the interpretation of our results since activation would have caused increased transcription of TFEB mRNA through a positive feedback loop^18^.

HPβCD has been used to both extract and deliver cholesterol to and from cellular membranes^19^. Thus, we then focused on a possible link between cholesterol and LF removal. Perhaps the best characterized system for therapeutic cholesterol manipulation by HPβCD is the extensive work conducted on NPC, where it has been shown that HPβCD is internalized through bulk-phase endocytosis. Once inside the late endosome/lysosome (LE/L), HPβCD overcomes the NPC1/2(−/−) disease-causing deficiency by helping release sequestered cholesterol from the lysosome to the cytosol, thus expanding the metabolically active cholesterol sink. This leads to both downregulation of cholesterol biosynthesis and upregulation of its efflux^1, 3, 6, 20^. For atherosclerosis, HPβCD-induced efflux within macrophages has been suggested as the mechanism which brings about symptom reduction^5, 21^.

**Figure 2:**
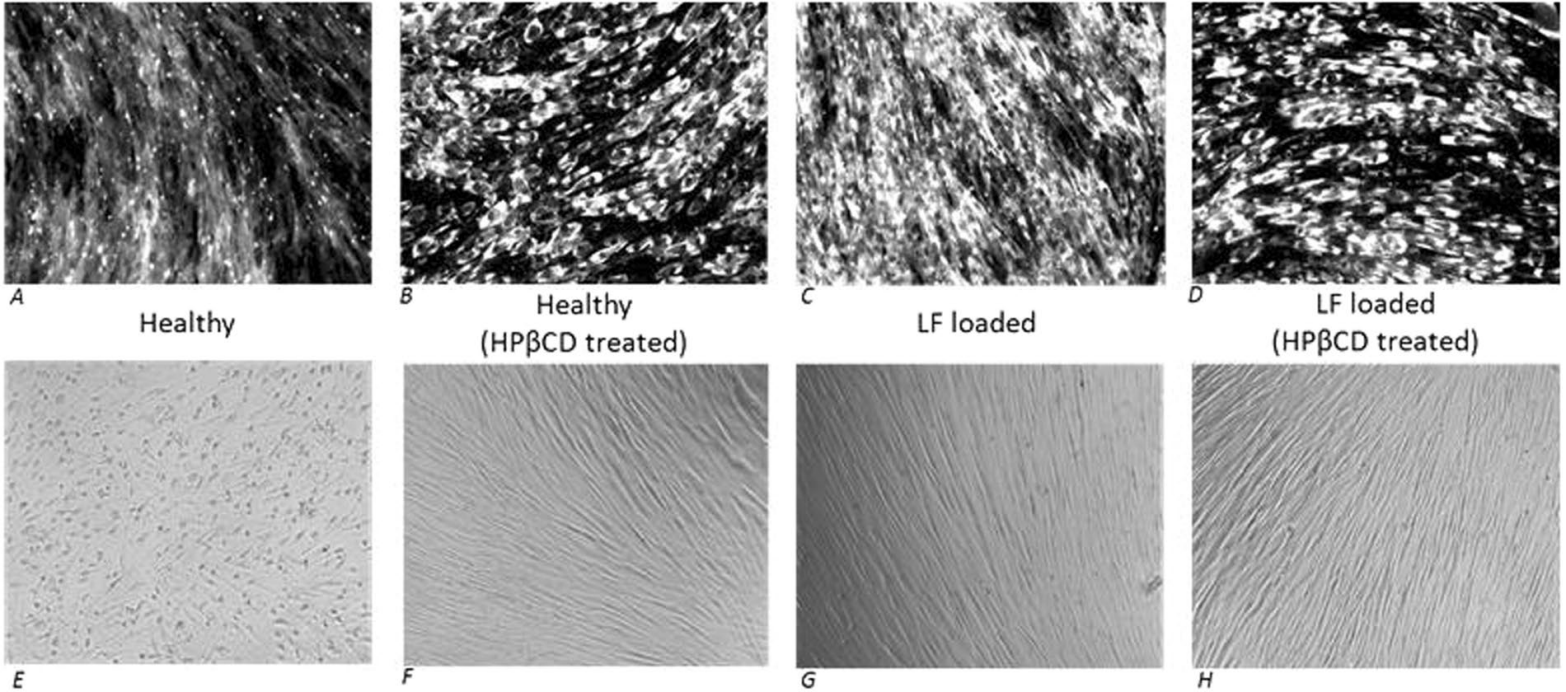
Long term HPβCD treatment increases cellular cholesterol. (**Top Row**) - Filipin staining shows HPβCD treatment, LF loading and the two combined each cause substantial cellular cholesterol increase relative to healthy cells. (**Bottom Row**) Cells with increased lysosome cholesterol composition are more resistant to apoptosis induced by the lysomotropic agent MSDH. Healthy confluent cells were killed by MSDH exposure (45 uM MSDH for 42 hours) while HPβCD treated cells and LF loaded cells survived with no signs of visual stress. Therefore, HPβCD and LF modulate lysosome membrane cholesterol composition in a manner consistent with that suggested by filipin staining.

For our system, filipin staining was used to assess cholesterol change. Exposure of cells to oxidative stress, which induces LF loading, resulted in a significant cholesterol increase (Fig. 2C). Interestingly, while HPβCD addition removes LF, no apparent reduction in cholesterol levels was observed between LF loaded cells (Fig. 2C) and HPβCD-treated LF-loaded cells (Fig. 2D). Surprising, healthy unaged cells also showed significant cholesterol increases upon HPβCD treatment (Fig. 2B). To verify the observed cholesterol changes we utilized the lysomotropic agent, O-methyl-serine dodecylamide hydrochloride (MSDH). Increased lysosomal cholesterol content decreases sensitivity to MSDH-induced cellular apoptosis^22^. Consistent with the filipin staining results, HPβCD–treated healthy cells, LF-loaded cells and HPβCD-treated LF-loaded cells (Fig. 2F–H) all showed increased resistance to MSDH relative to untreated controls (Fig. 2E).

Given LF is noted to perturb cholesterol metabolism, the observed increase in cholesterol for LF-loaded fibroblasts was not surprising^15, 23^. However, the increase in cholesterol observed with HPβCD-treated healthy cells is counterintuitive to most prior studies^24^. In fact, an HPβCD-induced cholesterol increase has been reported only once^25^. In that study no mechanistic explanation was explored and the cholesterol increase was observed only in young T-lymphocytes. In aged T-lymphocytes, HPβCD treatment elicited the commonly observed cholesterol decrease. For our data, with no apparent differences in filipin staining and MSDH resistance between LF loaded cells and HPβCD-treated LF loaded cells, the LF-removal mechanism by HPβCD was unclear. Thus, we used real-time PCR to quantify both cholesterol biosynthesis (*SREBP1, SREBP2, HMGCR, HMGCS, LDLr*) and efflux (*ABCA1, ABCG1, ABCG5, NPC1, NPC2*) gene expression.

**Figure 3:**
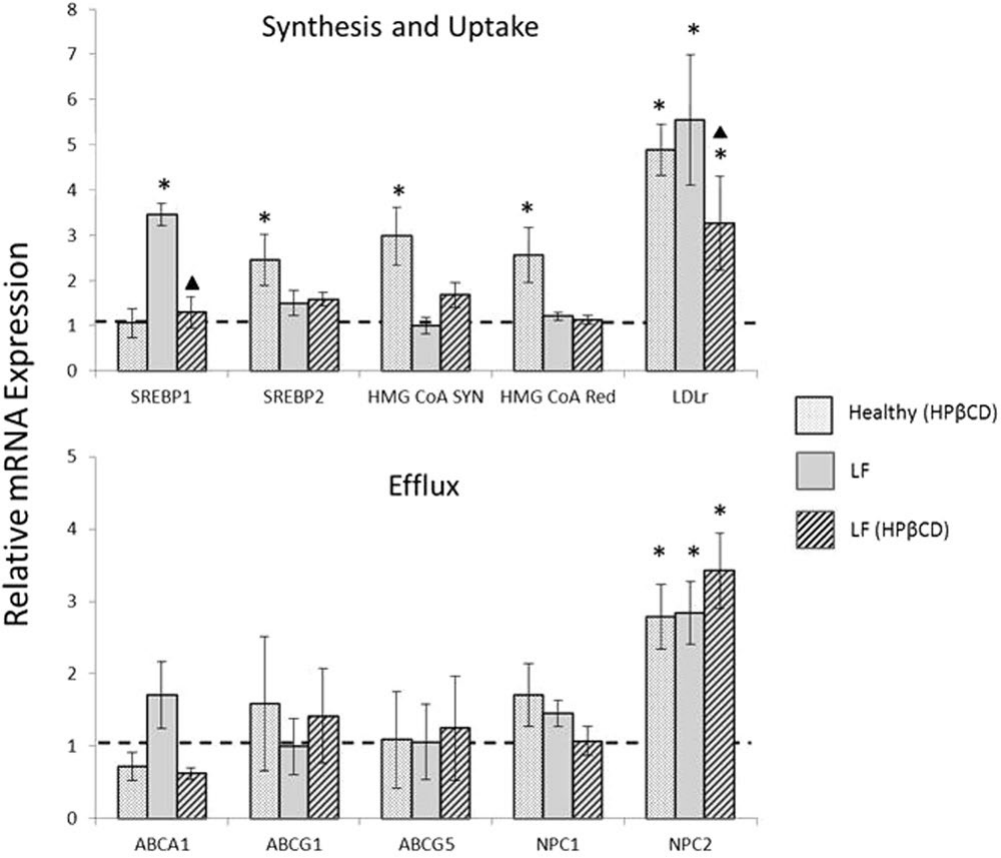
Response to HPβCD treatment varies according to initial cholesterol levels. mRNA levels for genes associated with cholesterol biosynthesis and uptake (**top graph**) and efflux (**bottom graph**). Asterisks (*) indicate statistical significance (*p* < 0.05) relative to healthy untreated cells. ^▲^Indicates statistical significance comparing LF-loaded HPβCD-treated cells relative to LF loaded cells. To be considered statistically significant the observed up or downregulation difference relative to control was ≥ 2.0 and the p-value < 0.05.

The observed cholesterol increases associated with LF loading coincide with overexpression of *SREBP1* and *LDLr* (Fig. 3). Cholesterol biosynthesis (in the form of *SREBP2, HMGCR, HMGCS*) was not activated. The addition of HPβCD to LF-loaded cells completely attenuated *SREBP1* overexpression back to control levels (from 3.5 to 1.3, P < 0.001), while *LDLr* expression was reduced by about 40% from 5.6 to 3.3 (P < 0.04). Finally, the cholesterol increases observed in HPβCD-treated healthy cells came from upregulation of both cholesterol biosynthesis and uptake genes. Specifically, *SREBP2, HMGCR and HMGCS* were all upregulated 2.5–3-fold (P < 0.001), while *LDLr* was upregulated nearly 5-fold (P < 0.001, Fig. 3). The expression of efflux genes remained unaffected for all test conditions, except for NPC2. However, since NPC2 was upregulated for all three test conditions (Fig. 3), we conclude that this is cholesterol-driven rather than an HPβCD-driven response.

**Figure 4:**
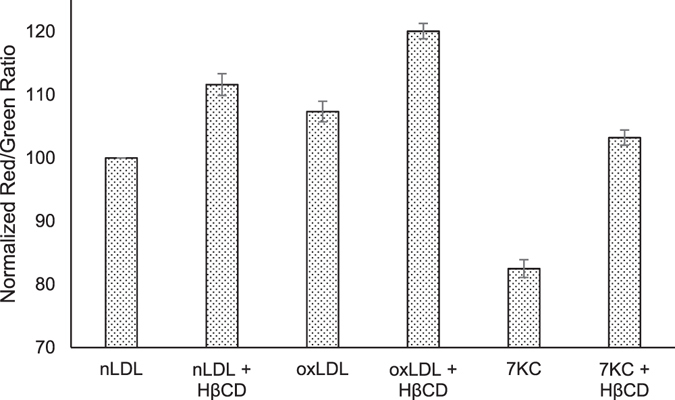
HPβCD attenuates the negative effects of cholesterol challenges. THP-1 cells were exposed to different LDL treatments over 7 days and then assessed for lysosomal membrane permeabilization using the acridine orange uptake assay. Acridine orange was excited at 488 nm and emissions recorded at 620 (red) and 520 nm (green). Lower red/green ratios are indicative of lysosomal instability. 7KC-LDL-induced lysosomal membrane permeabilization was significantly attenuated by HβCD (*p* < 0.05), which was added to samples on day 6. Flow cytometry signal intensities are reported as a percent of control the red/green ratio (normal LDL-treated). HβCD treatment improved the ratio in all cases, indicating decreased lysosomal permeabilization.

To investigate HPβCD’s influence on lysosome function, we exposed THP-1 cells to different LDL-loaded treatments over 7 days (Fig. 4) and then assessed for lysosomal membrane permeabilization using the acridine orange uptake assay. In all cases, HPβCD addition to each LDL treatment improved lysosome stability compared to the LDL treatments alone.

## Discussion

These results have profound therapeutic implications. Lipofuscin-like fluorophores associated with atherosclerotic plaques form on oxidized low density lipoprotein^13^. This is consistent with the observation that lipofuscin is often localized within lysosomes, the common endpoint for endocytosed LDL particles. Our data show upregulation of *LDLr* in both HPβCD-treated healthy cells as well as LF-loaded cells. However, only LF-loaded cells were subject to oxidation, supporting the hypothesis that oxidized LDL is a significant source for LF generation. The decrease in *LDLr* expression shown here with HPβCD addition to LF-loaded cells would limit oxidized LDL uptake and therefore explain the observed LF decrease. Furthermore, HPβCD decreases cholesterol accumulation in cultured NPC cells up to 3 days after HPβCD removal from the cell culture medium, which was attributed to HPβCD endocytosis^3^. Our results support this conclusion in two ways. First, similar to cholesterol, LF accumulation is also slowed six days after ceasing treatment with HPβCD (Fig. 1B). Second, HPβCD-driven *LDLr* repression would limit cholesterol uptake.

The observed upregulation in HPβCD-treated healthy cells is significant. First, the length of HPβCD treatment for our studies was 4 days. To date, the vast majority of publications have delivered cyclodextrin *in vitro* for 8 hours or less (short term)^24^, while animal studies typically use single or weekly injections^1, 18, 26^. Longer term HPβCD treatment, such as our study, exhausts cholesterol reserves likely by extracting cholesterol from the cell plasma membrane as well as mobilizing lysosomal cholesterol for the portion of HPβCD that is endocytosed. Intracellular membrane cholesterol levels are regulated by plasma membrane cholesterol levels^27^. Cells sensing this depletion respond by upregulating cholesterol biosynthesis and uptake (Fig. 3). In our system, efflux was not upregulated (Fig. 3), explaining the observed cholesterol increase in healthy cells. The data suggest that HPβCD’s therapeutic mode of action is based on nonspecific solubilization and extraction of cholesterol from the plasma membrane or within the lysosome, rather than promoting ABCA1/ABCG1 mediated efflux or mimicking NPC1/2 protein function as previously hypothesized^6^. In cholesterol-loaded cells, HPβCD treatment liberates cholesterol, which suppresses *LDLr*, and shifts cholesterol equilibrium in a manner that cholesterol flows away from the cell and lysosome.

In summary, our data corroborate HPβCD’s utility to treat age-related disorders where there is significant cholesterol accumulation. We also show that *LDLr* suppression is not limited to the NPC phenotype, but is likely a universal cellular response to HPβCD’s ability to expand the metabolically active cholesterol pool in cholesterol-loaded cells. This is the first report to demonstrate that *SREBP1*, a gene involved in regulation of LDLr and lipid metabolism, is attenuated with HPβCD treatment. This merits further exploration into how HPβCD may modify lysosome membrane composition, particularly given that lipid composition affects autophagy^28, 29^.

Overall, we advance understanding of the link between LF and oxidized LDLr. We also resolve a long-standing mechanistic debate about HPβCD’s mode of action: HPβCD nonspecifically binds and liberates cholesterol and oxysterols, thus shifting overall cellular cholesterol equilibrium away from the cell. We show that in healthy unaged cells, HPβCD treatment could cause unintended harm, by disturbing cholesterol equilibrium and upregulating cholesterol biosynthesis resulting in a net increase in cholesterol. Accordingly, the cholesterol content of the cell is a critical determinant of the effect caused by HPβCD treatment: from a well natured Dr. Jekyll-like cholesterol-lowering disease therapeutic, to a more sinister (unintended) cholesterol synthesis-inducing Mr. Hyde effect for healthy cells.

## Methods

### Cell Culture

Human skin fibroblasts (Coriell - GM00498), which are a commonly used model for the study of cyclodextrin and cholesterol flux as well as lipofuscin^3, 30^, were cultured in Eagle’s minimum essential medium, 10% fetal bovine serum, 1X glutaMAX, 20 IU/ml penicillin and 20 µg/ml streptomycin. Cells were incubated in humidified air with 5% CO_2_ at 37 °C. Unless otherwise stated, cells used for all experiments were between passages 15–24. Cells used to generate Fig. 1B were at passage 30. For all fibroblasts treated with HPβCD, final HPβCD concentration in cell culture solution was 7 mM. Cell viability after HPβCD treatment and LF loading was confirmed using a CCK-8 viability assay (Sigma).

### Lipofuscin generation, quantification and visualization

LF was generated and measured as previously described^31^. Fibroblasts were exposed to continuous oxidizing conditions (40 μM leupeptin, 45 μM FeCl_3_, and 10 μM H_2_O_2_) just prior to reaching confluency (≥90%) and for 10 days after. All media (including leupeptin, FeCl_3_, and H_2_O_2_) were replaced every two days during this 10 day period. For Fig. 1A, HPβCD was added to the media (or media + oxidizing reagents pending test condition) on days 6 thru 10. Each condition in Fig. 1A was repeated four times (N = 4). LF was measured at the end of day 10 using flow cytometry equipped with a PE filter (585/42 nm). Unlike the data in Fig. 1A, Fig. 1B results were obtained without the use of oxidizing reagents to promote the rapid development of LF. Rather, native LF (Fig. 1B) was obtained by serial passaging fibroblasts to passage 30 to simulate natural aging and to determine HPβCD’s ability to remove LF generated via natural cellular processes. At passage 30, cells were allowed to grow to confluency and separated into two groups: control (Fig. 1B left column) and test (Fig. 1B right column). Both groups were held at confluency for 12 days, with media replacement every 2^nd^ day. HPβCD treatment occurred on days 1–6 in the test group only. At the end of day 12, both groups were visualized for LF with a Nikon A1Rsi confocal microscope (100x, 640 nm). To determine HPβCD’s effect on lipopigment accumulation (Fig. 1C), fibroblasts (~P20) were grown to confluency and immediately exposed to continuous oxidizing conditions^31^ (the same conditions as used for Fig. 1A) in the presence and absence of HPβCD. An Olympus IX71 fluorescent microscope was utilized for lipopigment visualization. Images were taken at 100X with a FITC 540/40 filter, with constant exposure time and scaling between images to avoid transient effects of photobleaching and to ensure comparability.

### Cholesterol detection with filipin and O-methyl-serine dodecylamide hydrochloride (MSDH)

Filipin staining was performed using the Cholesterol Cell-Based Detection Assay Kit (Cayman Chemical 10009779) and protocol. Cells were immediately imaged at a constant exposure time to avoid confounding effects from photobleaching. The experiment was repeated for each condition three independent times, yielding the same results each time as shown in Fig. 2A–D. Images selected for apoptosis was induced by exposing cells to the lysomotropic detergent *O*-methyl-serine dodecylamide hydrochloride (MSDH), kindly provided by Hanna Appelqvist of Linkoping University. MSDH (45 µM final concentration, 42 hr treatment length) was added to confluent fibroblasts, which had previously been subject to continuous oxidizing conditions (the same conditions as used for Fig. 1A and C) to promote LF generation and/or HPβCD treatment for 7 continuous days after reaching confluence.

### Real Time PCR

*PCR*— Fibroblasts were grown to confluency and immediately exposed to continuous oxidizing conditions^30^ in the presence and absence of HPβCD. On day 4 of treatment total RNA was extracted using Trizol. RNA was then DNAse I digested and precipitated using 3 volumes of 100% ethanol mixed with 0.1 volume sodium acetate (3 M, pH 5) followed by subsequent 70% ethanol washes. Total RNA was quantified using NanoDrop. cDNA was generated using the RevertAid RT First Strand cDNA Synthesis Kit (Thermo). Quantitative PCR reactions were performed in a CFX96™ Real-Time PCR detection system (Bio-Rad) using cDNA, SYBR Green™, and the appropriate primers (Table S1). Samples were heated for 10:00 min at 95 °C followed by 40 cycles of amplification with 15 s at 95 °C, 30 s at 57 °C, and 45 s at 72 °C. Subsequent data analyses were conducted using Bio-Rad CFX manager software v3.1 (Bio-Rad). ΔC_T_ values were calculated using ACTB as the housekeeping gene. Relative mRNA expression level of a target gene was calculated as 2 exp[−(ΔC_T_ (treated cells) − ΔC_T_ (untreated cells))]. Each experimental condition was evaluated 6 times (N = 6).

### Acridine Orange Assay

Acridine orange uptake assays were used to assess lysosomal membrane permeabilization as previously described^32^. Briefly, THP1 cells (Sigma) were cultured in RPMI 1640 medium with 2 mM glutamine, 10% FBS, 20 IU/ml penicillin and 20 µg/ml streptomycin. Prior to plating, cells were centrifuged and resuspended in serum-free media containing 0.1% BSA for 24 h. 1% FBS to was then added to the cultures, and the cells were plated at 2.5 × 10^5^ in a 6-well plate. LDL treatments (nLDL, oxLDL, and 7KC-LDL) were added at 100 μg protein/mL. Media was swapped every two days, and cells were allowed to differentiate for six days. 0.9% HPβCD was then added for 24 h. Following treatments, cells were exposed to acridine orange (5 µg/ml) for 15 min at 37 °C. Cells were collected and resuspended in 500 µl PBS. Red (595 LP and 610/20) and green fluorescence (FITC), indicating acridine orange presence in lysosomes or the cytoplasm, respectively, was assessed by flow cytometry (LSRFortessa, BD Biosciences) using excitation at 488 nm.

### Preparation of modified LDL

Oxidized LDL (oxLDL) was prepared by dialysis with 20 μM CuSO_4_ in 4 L of 0.9% NaCl. LDL (Lee Bio) was pipetted into a Slide-A-Lyzer cassette (Thermo Scientific) and then dialyzed at 37 °C for 5 h. To stop the reaction, the oxLDL was then dialyzed twice against 0.9% NaCl containing 1 mM EDTA for 8 h. 7KC-loaded LDL was prepared by combining LDL with 7KC dissolved in ethanol, to give a final concentration of 2.4 mM 7KC with an ethanol concentration less than 2.6% (v/v). The solution was then incubated at 37 °C with gentle shaking for 6 h. All LDL stocks were purified by ultracentrifugation using a NaBr density gradient, followed by dialysis with 0.9% NaCl containing 1 mM EDTA. Protein concentrations were estimated based on absorbance at 280 nm, and the samples were concentrated by centrifugal filtration (7 kDa cutoff). TBARS was measured to determine the extent of oxidation, and 7KC loading was estimated after lipid extraction by HPLC using absorbance at 235 nm.

### Statistical Analyses

All data are presented as mean+/−S.D. Statistical significance was calculated using one-way analysis of variance (ANOVA).

## Electronic supplementary material

2-Hydroxypropyl-beta-cyclodextrin (HPβCD) reduces age-related lipofuscin accumulation through a cholesterol-associated pathway
