# Supplementary material for: 2-Hydroxypropyl-beta-cyclodextrin (HPβCD) reduces age-related lipofuscin accumulation through a cholesterol-associated pathway
Source: Sci Rep. 2017 May 19;7:2197. doi: 10.1038/s41598-017-02387-8 (PMC5438378; doi:10.1038/s41598-017-02387-8)
Supplement: Supplementary file 1 — 2-Hydroxypropyl-beta-cyclodextrin (HPβCD) reduces age-related lipofuscin accumulation through a cholesterol-associated pathway [file 41598_2017_2387_MOESM1_ESM.pdf]

Supplementary Materials for

2-hydroxypropyl-beta cyclodextrin (HPβCD) reduces age-related lipofuscin accumulation through a cholesterol-associated pathway

Jason Gaspar, Jacques Mathieu and Pedro Alvarez.

Rice University
Dept of Civil and Environmental Engineering
MS-519
6100 Main Street
Houston, TX 77005

correspondence to: alvarez@rice.edu

This PDF file includes:

Fig. S1
Table S1
References for Table S1

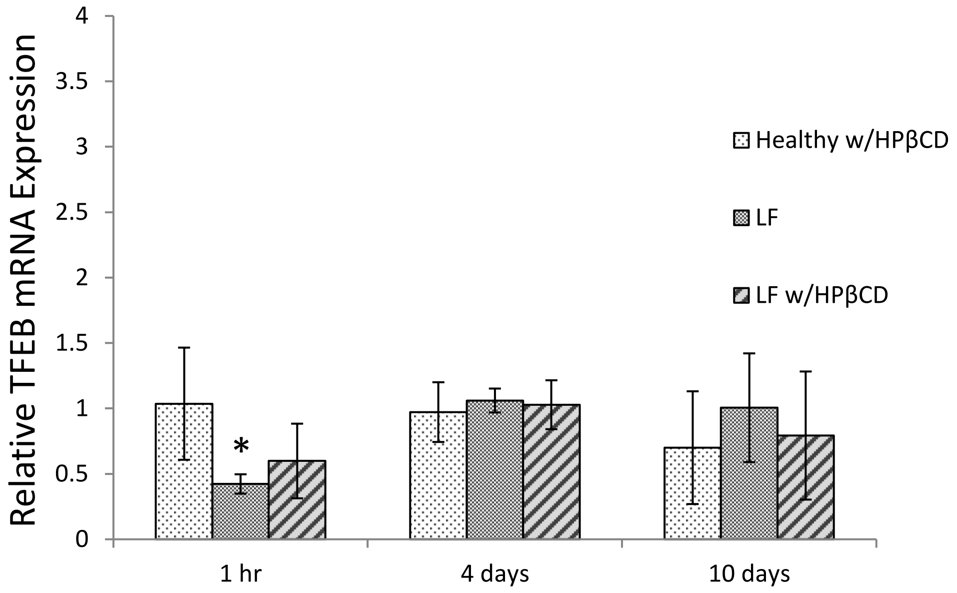

Fig S1. Lipofuscin removal is independent of lysosome biogenesis. mRNA levels of HPβCD-treated healthy and LF loaded cells over different time points. Asterisks (*) indicates statistical significance relative to healthy untreated cells (downregulation > 2, $p < 0.05$, $N \geq 5$)

Table S1. Oligonucleotide Sequences for real time PCR analysis

	**Forward Primer**	**Reverse Primer**	**Reference**
ACTB	GATGACCCAGATCATGTTTGA	ATAGCACAGCCTGGATAGC	-
TFEB	CCAGAAGCGAGAGCTCACAGAT	TGTGATTGTCTTTCTTCTGCCG	1
SREBP1	GCAAGGCCATCGACTACAT	GGTCAGTGTGTCCTCCA	2*
SREBP2	AGGAGAACATGGTGCTGA	TAAAGGAGAGGCACAGGA	2
HMGCS	GACTTGTGCATTCAAACATAGCAA	CTGTAGCAGGGAGTCTTGGTACT	3
HMGCR	TACCATGTCAGGGGTACGTC	CAAGCCTAGAGACATAATCATC	4
LDLR	CAATGTCTCACCAAGCTCT	TCTGTCTCGAGGGGTAGCT	5
ABCA1	GCACTGAGGAAGATGCTGAAA	AGTTCCTGGAAGGTCTTGTTCAC	2
ABCG1	CAGGAAGATTAGACACTGTGG	GAAAGGGGAATGGAGAGAAGA	2
ABCG5	ACCCAAAGCAAGGAACGG	CAGCGTTCAGCATGCCTG	2*
NPC1	CTTAGTGCAGGAACTCTGTCC	TCCACATCACGGCAGGCA	6*
NPC2	GGTTTGTCTTGTGACCGC	AGGAATGTAGCTGCCAGG	6

*Indicates primer was shortened from original published version

References for Table S1

1. M. Sardiello *et al.*, *Science* **325**, 473 (2009).
2. Y. K. Adlakha *et al.*, *Cell Death & Disease* **4**, e780 (2013).
3. S. Miyata, J. Inoue, M. Shimizu, R. Sato, *Bioscience, biotechnology, and biochemistry* **80**, 1006 (2016).
4. C.-C. Chen, T.-Y. Liu, S.-P. Huang, C.-T. Ho, T.-C. Huang, *Cellular Signalling* **27**, 2182 (2015).
5. Y. Chen, M. Hughes-Fulford, *Intl. J. of Cancer* **91**, 41 (2001).
6. J. E. McLaren *et al.*, *J. of Immunology* **185**, 1222 (2016).
